# Backscatter from therapeutic doses of ionizing irradiation does not impair cell migration on titanium implants in vitro

**DOI:** 10.1007/s00784-023-05128-6

**Published:** 2023-07-06

**Authors:** Lisa Printzell, Janne Elin Reseland, Nina Frederike Jeppesen Edin, Jan Eirik Ellingsen, Hanna Tiainen

**Affiliations:** 1 Department of Prosthodontics, Institute of Clinical Dentistry, Faculty for Dentistry, University of Oslo, PO box 1109, 0317 Blindern, Oslo Norway; 2 Department of Biomaterials, Institute of Clinical Dentistry, Faculty for Dentistry, University of Oslo, Oslo, Norway; 3 Department of Physics, Faculty of Mathematics and Natural Science, University of Oslo, Oslo, Norway

**Keywords:** Radiation backscatter, Titanium implants, DNA damage, Cell migration, Mesenchymal stem cells, Osteoblasts

## Abstract

**Objective:**

The influence of radiation backscatter from titanium on DNA damage and migration capacity of human osteoblasts (OBs) and mesenchymal stem cells (MSCs) may be critical for the osseointegration of dental implants placed prior to radiotherapy. In order to evaluate effects of radiation backscatter, the immediate DNA damage and migration capacity of OBs and MSCs cultured on titanium or plastic were compared after exposure to ionizing irradiation.

**Materials and methods:**

Human OBs and MSCs were seeded on machined titanium, moderately rough fluoride-modified titanium, or tissue culture polystyrene, and irradiated with nominal doses of 2, 6, 10, or 14 Gy. Comet assay was performed immediately after irradiation, while a scratch wound healing assay was initiated 24 h post-irradiation. Fluorescent live cell imaging documented the migration.

**Results:**

DNA damage increased with higher dose and with backscatter from titanium, and MSCs were significantly more affected than OBs. All doses of radiation accelerated the cell migration on plastic, while only the highest dose of 10 Gy inhibited the migration of both cell types on titanium.

**Conclusions:**

High doses (10 Gy) of radiation inhibited the migration capacity of both cell types on titanium, whereas lower doses (2 and 6 Gy) did not affect the migration of either OBs or MSCs.

**Clinical relevance:**

Fractionated doses of 2 Gy/day, as distributed in conventional radiotherapy, appear not to cause severe DNA damage or disturb the migration of OBs or MSCs during osseointegration of dental implants.

**Supplementary Information:**

The online version contains supplementary material available at 10.1007/s00784-023-05128-6.

## Introduction

Oral sequelae of radiotherapy (RT) are mutilating to head and neck cancer patients [1]. Preradiotherapy dental extractions are necessary for most of these patients [2], which may affect their appearance, impair their ability to speak and chew, and have an overall negative impact on the patients’ health-related quality of life [3, 4]. Temporary removable dentures are experienced as painful and they do not fulfill their desired functional purpose during the first 6 months after RT [5]. Compared to conventional complete- or partial-dentures, implant retained prostheses are recognized to be superior regarding masticatory performance, patients’ satisfaction, and quality of life [6, 7]. Despite the impaired healing capacity of irradiated bone, these major benefits make dental implants a feasible and valued treatment for many patients undergoing RT [8–11]. Thus, early permanent prosthetic reconstructions should be prioritized to help restore oral function to an acceptable level for the well-being of head and neck cancer patients [12].

Clinicians are increasingly recognizing the benefits of installing implants in head and neck cancer patients immediately after removal of teeth, prior to RT (primary placement) [13, 14], rather than the traditional delayed installation during the post-treatment follow-up (secondary placement) [15] which postpones the oral rehabilitation for years. In addition to providing these patients with functional prosthetic reconstructions as early as possible, survival rates of primary placed dental implants are comparable to those with secondary placement [13, 16]. Nevertheless, oral rehabilitation of head and neck cancer patients with the use of dental implants lacks consensus and recommendations regarding the timing of the surgery.

One central clinical concern towards primary placement of dental implants has been the effects of radiation backscatter from titanium, resulting in a higher dose of radiation to the tissues surrounding the implant [17, 18]. In the absence of conclusive clinical evidence, the question whether radiation backscatter impairs the osseointegration process of implants placed prior to RT to a larger extend than the reduced healing capacity of previously irradiated bone in the case of secondary placement still persists. Therefore, more studies are needed to identify the cellular effects of radiation backscatter, and thereby, proceed towards validation of primary placement of dental implants in patients planned for RT.

Ionizing radiation may cause either direct disruption of atomic structures in DNA molecules or indirectly affect it by cleavage of water molecules that form free water radicals, which in turn may ionize DNA [19]. Radiation-induced DNA damage activates cell responses depending on the severity of the lesion: either an apoptotic signalling cascade eliminates the cell or the cell will stop dividing while DNA repair enzymes try to repair the damage [20–22]. The goal of RT is always to maximize the genotoxic effect in the tumor, while limiting radiation injury to surrounding normal tissues [19]. In conventional RT, this is achieved by distributing the radiation in dose fractions, typically 2 Gy per day over 5–7 weeks [23].

The osseointegration of a titanium implant is a process similar to wound healing [24, 25]. Since cell migration is a rate-limiting event in wound healing, in vitro analysis of this ability is important in order to evaluate the osseointegration potential. Interestingly, low-level laser irradiation has shown to stimulate a range of cellular processes and promote soft and hard tissue repair in rats [26, 27]. However, no study has yet investigated the effects of *therapeutic* doses of ionizing irradiation on the migration of cells involved in the osseointegration process. In this study, primary human OBs and MSCs were exposed to clinically relevant doses of γ-radiation to assess the biological effects caused by the radiation reinforced with backscatter from titanium implant surfaces. For this purpose, radiation-induced single and double-strand DNA damage was determined by the comet assay and the cell migration capacity was evaluated in a wound healing scratch assay.

## Material and methods

### Test surfaces

Commercially pure (cp) minimally rough grade 2 titanium (Ti) and moderately rough fluoride-modified grade 4 titanium (TiF) disks with a diameter of 6.25 mm and height of 1.95 mm were used as test surfaces, with tissue culture polystyrene (TCP) serving as a control surface for assessing the effect of radiation backscatter. The machined Ti-surfaces were washed first in trichloroethylene for 15 min, then in absolute ethanol (100%) in an ultrasonic bath for 15 min, before being sterilized in an autoclave at 135 °C for 20 min. The TiF-surfaces were manufactured by Dentsply Sirona implants according to the same protocol as the commercially available implant surface Osseospeed. The disk surface was grit-blasted with titanium dioxide (TiO_**2**_) microparticles, followed by an initial etching in concentrated nitric acid (HNO_3_) and sodium hydroxide (NaOH), respectively, and a final etching step in 0.1 M hydrofluoric acid (HF) before being pre-mounted on carriers in sealed containers and sterilized by β-irradiation (Osseospeed, Dentsply Sirona implants, Mölndal, Sweden).

### Cell culture

Commercially available primary human osteoblasts (OBs; batch#: 0000426160) and human mesenchymal stem cells (MSCs; batch#: 0000684888) (Lonza, Walkersville, MD, USA) were routinely cultured at 37 °C/5% CO_2_ in osteoblast basal medium (C-27010) supplemented with Osteoblast Growth Medium SupplementMix (C-39615) (Promocell, Heidelberg, Germany), or in human Mesenchymal Stem Cell Growth BulletKit Medium (MSCGM catalog no. PT-3001) (Lonza) respectively, both containing L-glutamine, gentamicin sulfate-amphotericin (GA), and 10% fetal bovine serum (FBS). Cells were subcultured before reaching confluence using trypsin/EDTA (Sigma-Aldrich). Trypan blue staining was used to determine total and viable cell number. Cells were seeded at a density of 3.5 × 10^3^ cells/well on TCP and 4.5 × 10^3^ cells/disk on Ti and TiF disks in 96-well plates for the comet assay, and at a density of 7.5 × 10^4^ cells/well or disk in 48-well plates for the wound healing assay and incubated overnight at 37 °C/5% CO_2_ prior to irradiation. Experiments were performed with cells at passages < 7 after isolation.

Following overnight incubation, the basal growth medium was changed and the cells on the different sample surfaces were exposed to various doses of γ-radiation. Irradiation times for nominal doses of 2, 6, 10, and 14 Gy were calculated considering the source-to-plate distance of 70 cm and the decay of the ^60^Co source (Theratron 780-C, MDS Nordion, Ontario, Canada). A dose of 10 Gy delivered on a titanium surface is equivalent to a dose of 14 Gy delivered on TCP, taking the additional radiation backscatter into account [28]. Therefore, the highest dose of 14 Gy was only used for cells cultured on TCP. To maintain the cell medium temperature at 37 °C, a hollow perspex plate transfused with circulating pre-heated water (Grant Instruments, Cambridge, England) was used throughout the irradiation procedure. Dose measurements were achieved by thermoluminescence dosimetry (TLD) (TLD-100; Harshaw TLD Bicrom, Solon, OH, USA). The irradiation was performed at The Norwegian Radium Hospital, Oslo University Hospital.

### Comet assay

An alkaline single cell electrophoreses comet assay was used to quantify immediate DNA damage in OBs and MSCs exposed to various doses of irradiation on the different sample surfaces. The cells were kept on ice whenever practically possible throughout the entire sample preparation process to minimize immediate rejoining of strand breaks induced by the radiation.

Immediately after the irradiation, the cells were detached from sample disks or wells with trypsin/EDTA and resuspended in 225 µl culture media. After centrifugation (5 min at 200 × *g*), the supernatant was removed and the cell pellet was mixed in 26 µl of 1% (w/v) low melting point agarose. The agarose-cell suspension was divided into two equal drops of 12 µl and placed on an agarose-coated microscope slide. Slides were stored at 4^○^C for 10 min for complete gelation. A positive control (unirradiated cells on TCP) was placed in a slide holder filled with cold 3% H_2_O_2_ for 5 min. All slides were then placed in slide holders filled with a cold lysis solution (2.5 M NaCl, 100 mM EDTA, 10 mM Tris–HCl, pH = 10, 1% triton X-100) and stored at 4^○^C for 1 h. All slides were placed in an alkaline electrophoresis solution (300 mM NaOH, 1 mM EDTA, pH > 13) for 20 min at 4^○^C for DNA unwinding before electrophoresis was run for 30 min at 25 V and 320 mA at 4^○^C. Slides were neutralized with cold PBS and d-H_2_O for 10 min each and dried overnight at 4^○^C. After drying, the slides were stored at room temperature. For TCP samples, the comet assay was also performed following 24-h incubation after the irradiation using the same protocol for sample preparation.

Nuclei were stained with 0.1 µg/ml SYBR gold (Invitrogen, Eugene, OR, USA) in darkness at room temperature for 15 min shortly before imaging. Slides were imaged using a fluorescence microscope with 4 × objective (Olympus IX70, Olympus, Tokyo. Japan). At least 50 nuclei per droplet (100 per sample; *n* = 3) were imaged and analyzed using the OpenComet plugin (v1.3) for ImageJ. Results of the comet assay are presented as %DNA in tail [29] and as relative DNA damage representing %DNA in tail normalized to the unirradiated sample on corresponding surface (C^−^) and the positive TCP control (C^+^; 3% H_2_O_2_) according to the following equation:

$$\mathrm{Relative DNA damage }\left(\mathrm{\%}\right)= \frac{\mathrm{Sample}-{\mathrm{C}}^{-}}{{\mathrm{C}}^{+}-{\mathrm{C}}^{-}}\times 100$$

### Wound healing assay

Scratch assay was performed to evaluate the migration capacity of human OBs and MSCs growing on Ti, TiF, and TCP after being exposed to single doses of ionizing γ-irradiation. Following 24 h of incubation at 37 °C/5% CO_2_ after the irradiation, the basal growth medium was changed to a serum reduced medium with only 1% FBS to reduce cell proliferation [30]. Two hours later, the cell monolayers were stained with CellTracker Green CMFDA (Invitrogen, Eugene, OR, USA). Scratches formed like a cross were then scraped on each TCP sample using a 200-μl pipette tip, followed by washing with PBS. The cross enabled identification and imaging of the exact same area at each timepoint throughout the experiment (0 h, 6 h, 12 h, 24 h, 30 h, 48 h, 60 h) using an Olympus IX70 fluorescence microscope with 4 × objective (Olympus, Tokyo, Japan). On Ti and TiF-surfaces, the scratch assay was performed with some modifications. Due to the opacity of the Ti and TiF disks, the adherent cells were stained with CellTracker Green CMFDA and disks monitored in a confocal laser scanning microscope before the irradiation to ensure all included samples were covered with a healthy monolayer of OBs or MSCs. Following 24-h incubation at 37 °C/5% CO_2_ after the irradiation, cells were stained with CellTracker Green CMFDA again and monitored before a scratch was scraped in a straight line through the cell monolayer on each included disk using a 200-μl pipette tip while holding the disk in place with a tweezer. The scratch was performed in a Petri dish with preheated medium (1% FBS), and then immediately placed in an incubator attached to an upright Leica SP8 confocal laser-scanning microscope with air objective lens HC APO CS 10 × /0.40 (Leica, Wetzlar, Germany) to acquire the baseline image (0 h). With the format of 1024 × 1024, the whole scratch/diameter of the disk was captured in 3 images, which enabled us to find the same area on each disk at each subsequent timepoint (24 h, 48 h, and 72 h).

Upon completion of the experiment, images from each timepoint were analyzed with respect to the gap width and presented as % of total “gap closure” (GC) as illustrated in Fig. S1 in the online supplementary information. Mean measures of each disk from the different timepoints (24 h, 48 h, 72 h) were normalized to 0 h (baseline). The gap edges (measure points) were defined as the recognition of the leading cells on each side of the gap, with maximum 5 countable cells inside the gap that were not already there at 0 h. Additionally, the “gap area filled with cells” (GFC) was estimated and defined as the % of original gap-area evenly filled with cells, without focus on cell density (Fig. S1, Online Resource). ImageJ software (National Institutes of Health and the Laboratory for Optical and Computational Instrumentation) was used for image processing and analysis [31]. The same operator evaluated all images 3 times, on 3 different days, to insure intra-rater reliability. For illustration purposes, background fluorescence was removed from the presented microscopy images of the TCP-surface using Adobe Photoshop 2020. All qualitative and quantitative image analyses were performed before the images were edited.

### Data analysis

The effects of irradiation (2, 6, 10, and 14 Gy) on DNA damage and migration capacity of human OBs and MSCs growing on three different surfaces (TCP, Ti, TiF) were graphically presented using Sigmaplot (V 14.0 for Windows; Systat, Chicago, IL, USA). Data from the comet assay is presented as mean values ± standard error of the mean (SEM; *n* = 3). Differences between groups were assessed using one-way ANOVA followed by pairwise multiple comparison using Holm-Sidak method with significance level set to *p*-values ≤ 0.05. The data was checked for normality and equal variance using Shapiro–Wilk test and Brown-Forsythe test, respectively. Data from the scratch assay is graphically presented using mean values ± standard deviation (SD).

## Results

### DNA damage

**Fig. 1:**
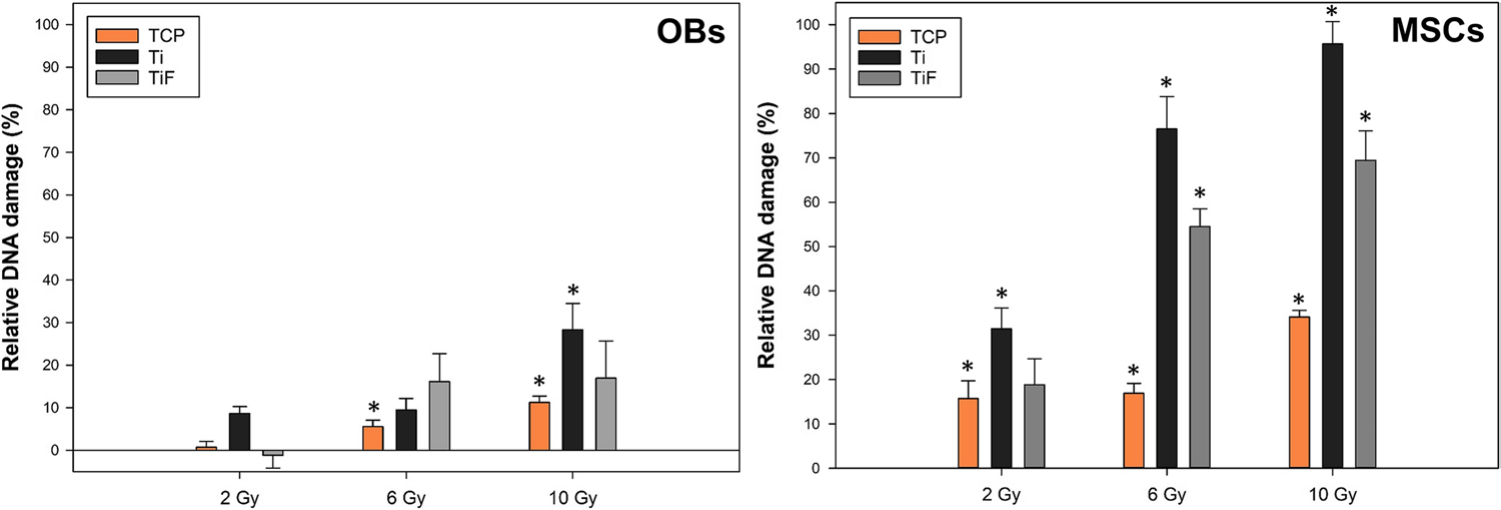
Relative DNA damage with %DNA in tail normalized to the unirradiated samples on corresponding surface (C^−^) and positive TCP control (C^+^; 3% H_2_O_2_), of primary human osteoblasts (OBs) and human mesenchymal stem cells (MSCs) immediately after irradiation with 0, 2, 6, and 10 Gy, while growing on tissue culture polystyrene (TCP), minimally rough machined titanium (Ti), or moderately rough fluoride-modified titanium (TiF) (*n* = 3). Data are presented as mean ± SEM. * *p* ≤ 0.05 compared to corresponding 0 Gy control (C.^−^)

The relative DNA damage in primary human OBs and MSCs immediately (0 h) after exposure to ionizing irradiation (2, 6, 10 Gy) is presented in Fig. 1. Increasing radiation doses induced increasing amounts of DNA damage in both cell types, but MSCs were significantly more affected by radiation than the OBs after all tested doses (*p* < 0.05), and the highest levels of DNA damage were observed in MSCs irradiated on the titanium surfaces, especially the Ti-surface (Fig. 1).

A significantly increased amount of DNA damage was found in unirradiated MSCs on TiF and to a lesser degree on Ti compared to TCP. A similar, but less pronounced and not statistically significant, trend was observed for unirradiated OBs (Fig. S2, Online Resource). After incubating the cells in 10% FBS for 24 h following the irradiation, no dose-dependent difference in DNA damage was observed in either the OBs or MSCs cultured on TCP (Fig. S3, Online Resource). However, the high DNA in tail values observed for all groups following 24-h incubation, including the unirradiated control group, limits the conclusions that could be drawn based on this result.

### Wound healing assay

After irradiation, none of the Ti disks contained a confluent monolayer of MSCs. Consequently, the scratch wound healing assay was performed with OBs on TCP, Ti, and TiF, and with MSCs only on TCP and TiF.

**Fig. 2:**
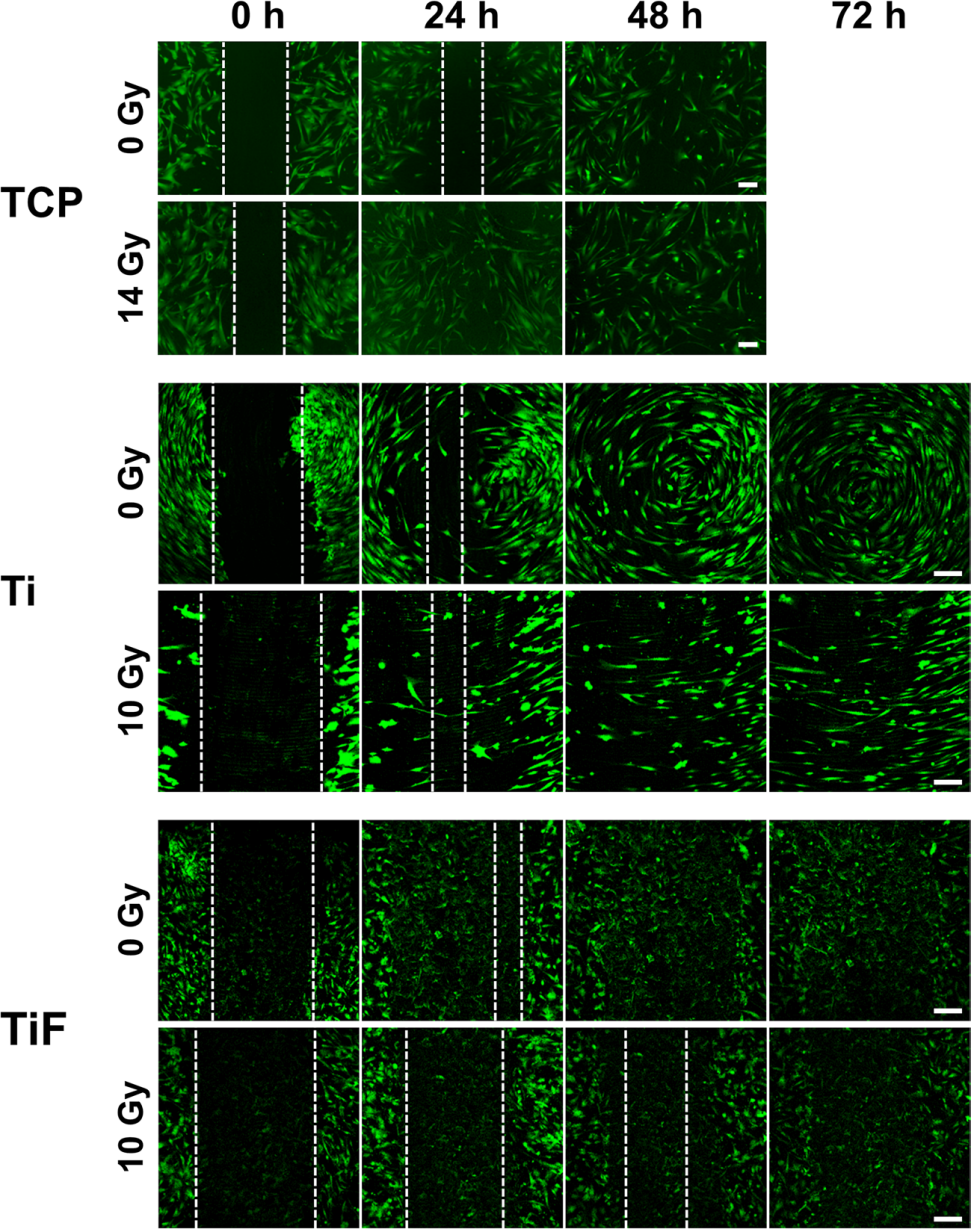
Representative images of primary human osteoblasts (OBs) migration and gap closure (GC) on tissue culture polystyrene (TCP), minimally rough machined titanium (Ti), and moderately rough fluoride-modified titanium (TiF), after no irradiation and 14 Gy (TCP) or 10 Gy (Ti + TiF), at the timepoints 0, 24, 48, and 72 h. As full gap closure was already observed at 48 h for all TCP samples, no imaging was performed at later timepoints. Scale bar: 200 µm

**Fig. 3:**
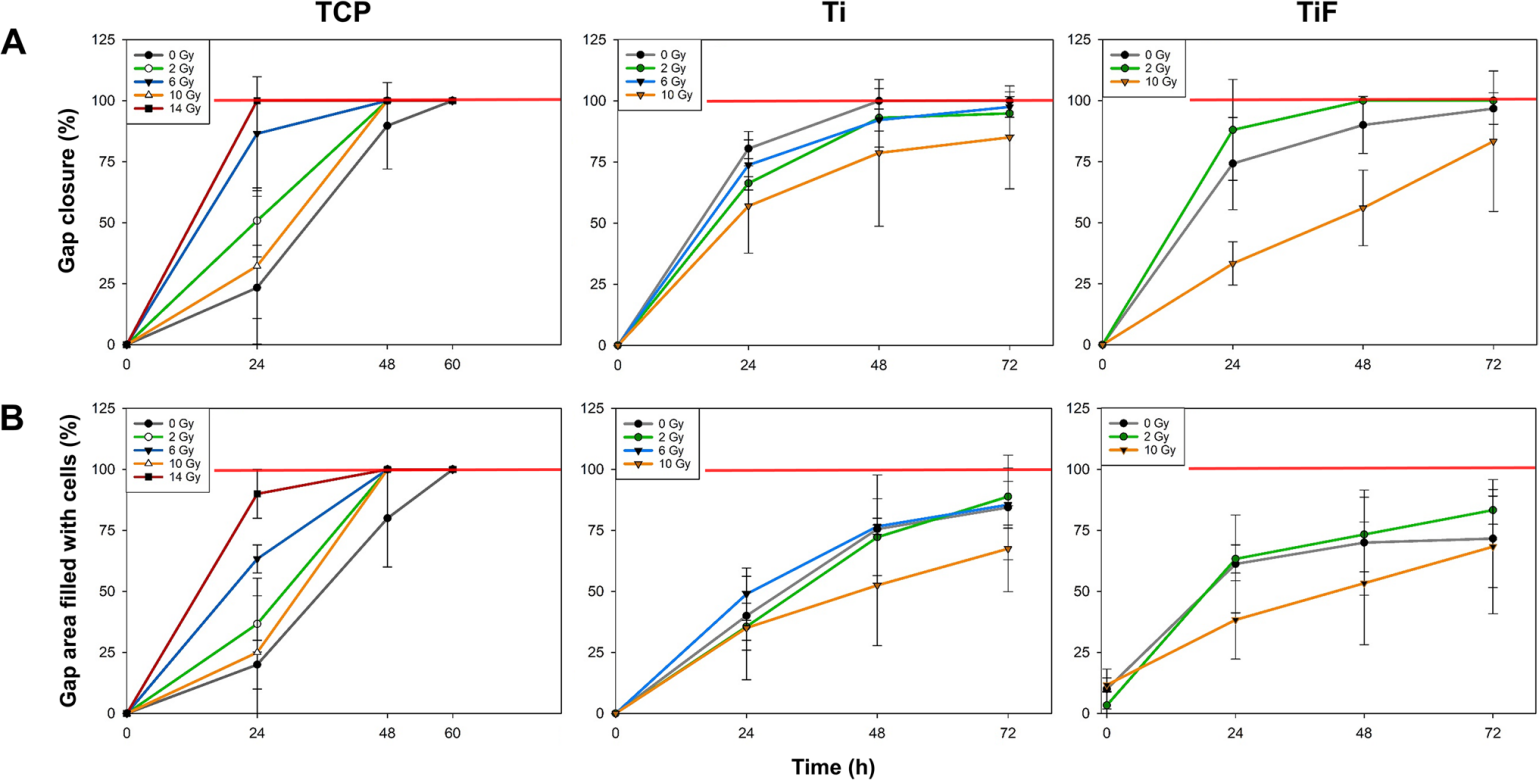
Graphical illustration of the quantified **A** gap closure (GC) and **B** gap-area evenly filled with cells (GFC) on tissue culture polystyrene (TCP) after irradiation with 0, 2, 6, 10, and 14 Gy (*n* = 3), on minimally rough machined titanium (Ti) after irradiation with 0 (*n* = 3), 2 (*n* = 3), 6 (*n* = 3), and 10 (*n* = 2) Gy, and on moderately rough fluoride-modified titanium (TiF) after irradiation with 0 (*n* = 4), 2 (*n* = 3), and 10 (*n* = 3) Gy. Scratches were performed 24 h post-irradiation and images were captured at the timepoints 0, 24, 48, and 60 h (TCP) or 72 h (Ti and TiF) after the scratch. Data represent mean values ± SD

**Fig. 4:**
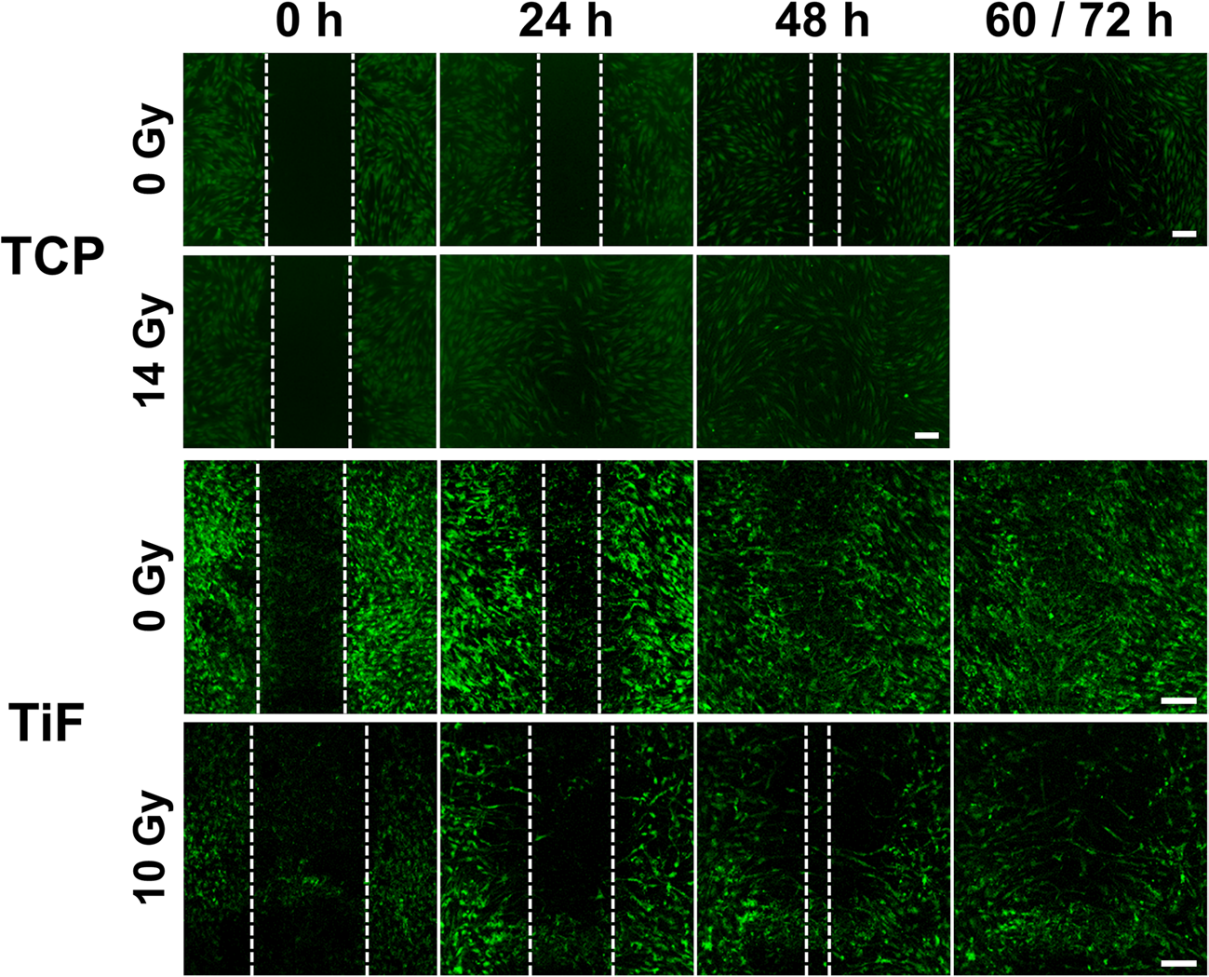
Representative images of human mesenchymal stem cells (MSCs) migration and gap closure (GC) on tissue culture polystyrene (TCP) and moderately rough fluoride-modified titanium (TiF), after no irradiation and 14 Gy (TCP) or 10 Gy (TiF), at the timepoints 0, 24, 48, and 60 h (TCP) or 72 h (TiF). As full gap closure was already observed at 48 h for the 14 Gy TCP samples, no imaging was performed at 60 h. Scale bar: 200 µm

**Fig. 5:**
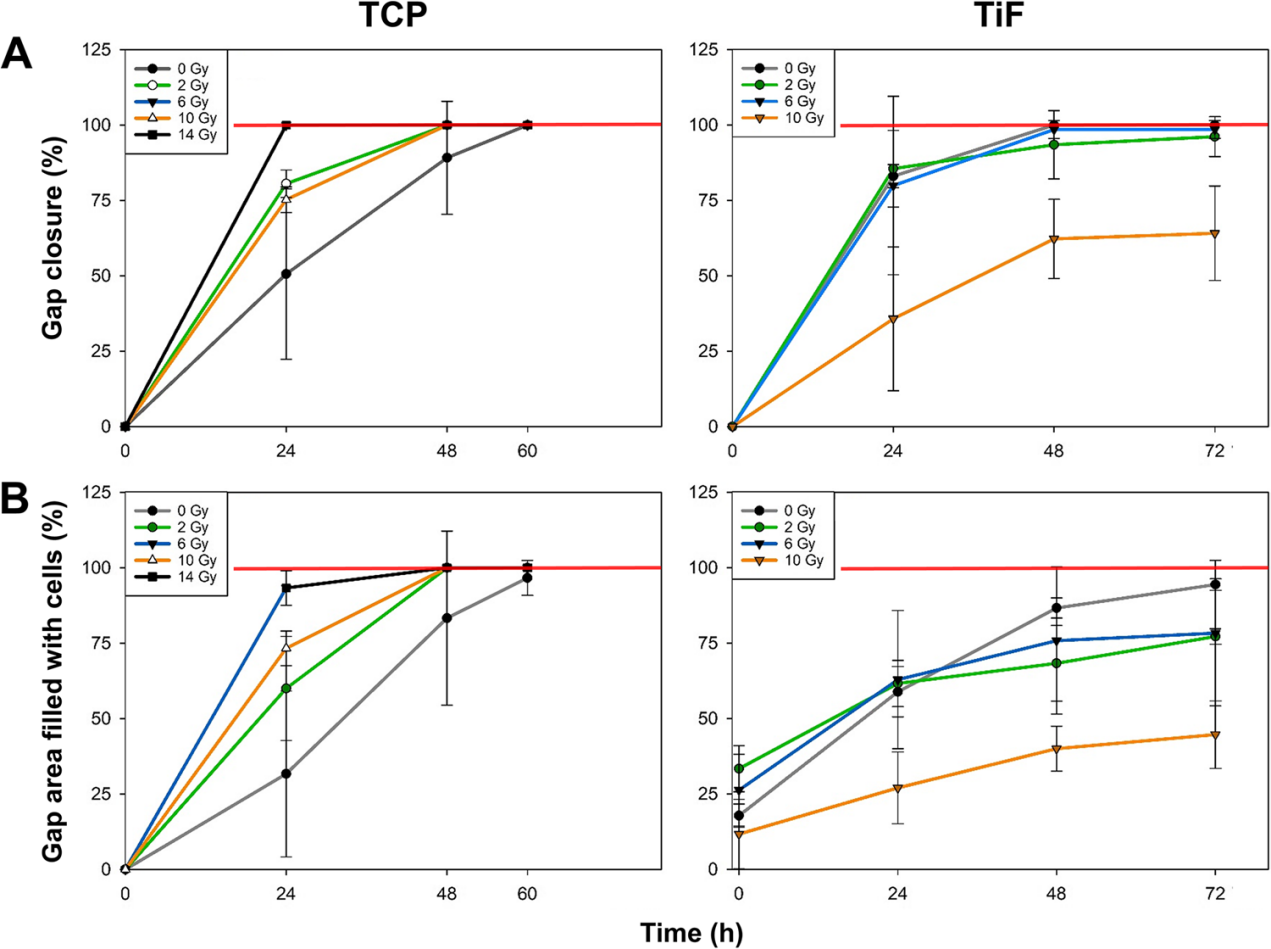
Graphical illustration of the quantified **A** gap closure (GC) and **B** gap-area evenly filled with cells (GFC) on tissue culture polystyrene (TCP) after irradiation with 0, 2, 6, 10, and 14 Gy (*n* = 3), and on moderately rough fluoride-modified titanium (TiF) after irradiation with 0 (*n* = 3), 2 (*n* = 3), 6 (*n* = 4), and 10 (*n* = 5) Gy. Scratches were performed 24 h after irradiation and images were captured at the timepoints 0, 24, 48, and 60 h (TCP) or 72 h (Ti and TiF) after the scratch. Data represent mean values ± SD

Irradiation with 14 Gy increased the migration rate of both OBs (Figs. 2 and 3) and MSCs (Figs. 4 and 5) cultured on TCP, resulting in a total gap closure (GC) within 24 h. In contrast, *unirradiated* OBs and MSCs on TCP migrated the slowest and did not reach GC until 60 h after the initial scratch.

On the titanium surfaces (Ti and TiF), only the highest dose of 10 Gy slowed down the migration rate of both OBs (Figs. 2 and 3) and MSCs (Figs. 4 and 5).

## Discussion

The comet assay is widely used for measuring DNA damage and repair in cells and tissues [32, 33], and the method has previously confirmed a linear relation between increased doses of ionizing irradiation and larger amounts of DNA damage in the exposed cells or organisms [29, 34, 35]. Correspondingly, this study showed increased DNA damage with increasing doses, but additional damage caused by radiation backscatter was only observed in the MSCs, especially from those irradiated on the minimally rough Ti-surface. In general, the MSCs were significantly more affected than the OBs immediately after irradiation, irrespective of dose and surface. In fact, OBs irradiated on the TiF-surface showed no significantly increased DNA damage after any doses.

The substantially higher DNA damage found in MSCs after irradiation on the Ti-surface could explain why none of these disks retained a confluent monolayer of MSCs 24 h post-irradiation. On the other hand, MSCs were sustained on the TiF-surface after all irradiation doses, even though this surface induced the highest DNA damage in *unirradiated* MSCs, equaling the total damage in MSCs on the two titanium surfaces (Fig. S2, Online Resource). Hence, the absence of irradiated MSCs on the Ti disks could perhaps be linked to inhibition of adhesion rather than severe DNA damage.

Several studies have shown increased cell adhesion to rougher titanium surfaces compared to smooth or polished titanium [36–38], but also microscale and nanoscale modifications of the titanium such as biomimetic physicochemical methods using proteins, peptides, and bioactive ceramics have shown to affect cell adhesion, spreading, and proliferation due to enhanced wettability of the surface [39, 40]. Accordingly, Li et al. found that irradiated osteoblast-like cells adhered stronger to a microarc oxidated titanium compared to a polished titanium, and surprisingly, also stronger than unirradiated controls [41]. However, another research group found that 5 and 10 days after irradiation, OBs attachment to both titanium and plastic decreased significantly [42]. In the present study, we experienced that both OBs and MSCs were difficult to remove from the TiF-surface with a pipette-tip, while on the Ti-surface, the entire cell sheet loosened in conjunction with the scratch in a pilot experiment. This may be explained by the higher surface roughness as well as higher wettability of the TiF surface compared to the Ti surface used in this study [43].

Based on a limited number of previous studies, OBs exposed to ionizing irradiation while cultured on titanium show a dose-dependent decrease in cell proliferation [41, 42, 44], while MSCs are known to be relatively radioresistant [28, 45]. Irradiation activates cell cycle checkpoints and DNA damage responses, but as revealed using the comet assay, early passage (2–3) MSCs are more resistant to irradiation and DNA damages induced by genotoxic agents than late-passage (> 10) MSCs [46]. Based on the knowledge that MSCs are quite radio-resistant, combined with the high DNA damage found immediately after irradiation, it seems like MSCs must inhere effective DNA repair mechanisms. Calini et al. showed that most DNA damage in mouse embryo fibroblasts exposed to high doses of irradiation (5 and 10 Gy) was repaired within 2 h, and the highest rejoining rate occurred with the highest dose of 10 Gy. Conversely, after a low dose of 1 Gy, no repair occurred within the first 2 h, but complete recovery was observed in all radiation groups after 24 h [34]. Another research group investigated the effects of γ-irradiation from a ^60^Co source with doses ranging from 1 to 50 Gy, on a large number of *A. aegypti* adult mosquitos. All radiation doses, except 1 Gy, exhibited a significant increase in DNA damage, with the highest levels observed 1 h post-irradiation. Total repair of damage occurred after 3 h in the 5 Gy group, after 6 h in the 10 Gy group, and after 12 h, no residual damage was found even in the 50 Gy group, although by then, heavily damaged cells may have been lost due to apoptosis [35]. Accordingly, we chose to initiate the scratch wound healing assay 24 h post-irradiation to ensure that all potential reversible DNA damage was repaired.

Even though the scratch assay is a simple, cost-effective, and very useful method to study cell migration in vitro [47], there are just a handful of such studies performed with cells growing on titanium [48, 49], and none of these is performed with cells exposed to ionizing irradiation. In this study, the migration capacity of OBs or MSCs growing on titanium was not altered by irradiation doses of 2 or 6 Gy, while a 10-Gy dose inhibited the migration, especially on the TiF-surface. The opposite was observed for both cell types on TCP, with increased migration after higher doses of irradiation. Although low-level laser irradiation and far-infrared irradiation have previously been shown to accelerate the migration of human OBs and rat bone marrow derived stem cells [50, 51], the considerably faster migration rate with full gap closure observed already after 24 h for both OBs and MSCs irradiated with a 14 Gy dose compared to 60 h for unirradiated cells on TCP was an unexpected finding. However, the results from the scratch assay support the assumption that most DNA damage observed in the cells immediately after irradiation was repaired before initiating the assay 24 h post-irradiation, except after 10 Gy when reinforced with backscatter from titanium. Based on theoretical calculations, backscatter from titanium increases the relative effective radiation dose by more than 40% 10–20 μm from the implant surface, resulting in a 10-Gy dose on Ti and TiF corresponding to 14 Gy on TCP [28]. However, the backscattered radiation also has lower energy, and therefore, higher linear energy transfer (LET) than the primary radiation from the ^60^Co source [52]. Higher LET is typically related to higher relative biological effectiveness of the radiation [53], which may explain both the increased DNA damage and the reduced migratory capacity of cells on the titanium surfaces compared to TCP exposed to theoretically corresponding radiation doses.

One limitation worth mentioning for the scratch assay is that proliferation needs to be inhibited to detect true cell migration. Avoiding serum in the cell culture medium is one way used to suppress cell proliferation [54]. Nonetheless, primary human cells need serum for the movement and overall survival, and therefore, totally serum-free medium is not recommended [30, 55]. Hence, using low serum concentrations as we did may inhibit, but not totally stop, cell proliferation. However, the clinical relevance of this study is not diminished even if the results may represent a combination of migration and proliferation, as ionizing irradiation also induces cell cycle delay and reduces cell proliferation [56].

Nevertheless, this is the first study to report in vitro migration of OBs and MSCs on a titanium implant surface after being exposed to ionizing irradiation with backscatter from titanium. The objective was to evaluate the effects of radiation backscatter on DNA damage and migration capacity of OBs and MSCs exposed to ionizing irradiation while growing on titanium. In conclusion, we found that radiation backscatter caused negative effects in form of increased DNA damage in MSCs immediately after irradiation, and reduced migration capacity of both OBs and MSCs after high doses of ionizing irradiation. However, low doses, as received in fractionated radiotherapy, do not seem to affect the migration of OBs or MSCs even when a dental titanium implant is generating radiation backscatter.

## Supplementary Information

Below is the link to the electronic supplementary material.

Supplementary file1 (PDF 353 KB)

## Data Availability

Data available on request from the authors.
