# Supplementary material for: Backscatter from therapeutic doses of ionizing irradiation does not impair cell migration on titanium implants in vitro
Source: Clin Oral Investig. 2023 Jul 6;27(9):5073–82. doi: 10.1007/s00784-023-05128-6 (PMC10492688; doi:10.1007/s00784-023-05128-6)
Supplement: Supplementary file 1 — Supplementary file1 (PDF 353 KB) [file 784_2023_5128_MOESM1_ESM.pdf]

Backscatter from therapeutic doses of ionizing irradiation does not impair cell migration on titanium implants in vitro

Supplementary information

Clinical Investigations in Dentistry

Lisa Printzell[1], Janne Elin Reseland[2], Nina Frederike Jeppesen Edin[3], Jan Eirik Ellingsen[1], Hanna Tiainen[2]

[1]Department of Prosthodontics, Institute of Clinical Dentistry, Faculty for Dentistry, University of Oslo, Oslo, Norway

[2]Department of Biomaterials, Institute of Clinical Dentistry, Faculty for Dentistry, University of Oslo, Oslo, Norway

[3]Department of Physics, Faculty of Mathematics and Natural Science, University of Oslo, Oslo, Norway

Corresponding author: lisa.printzell@odont.uio.no

Table of contents

Image analysis for cell migration assay S3
Fig. S1 .... S3
DNA damage S4
Fig. S2 .... S4
Fig. S3 .... S5
References S6

Image analysis for cell migration assay

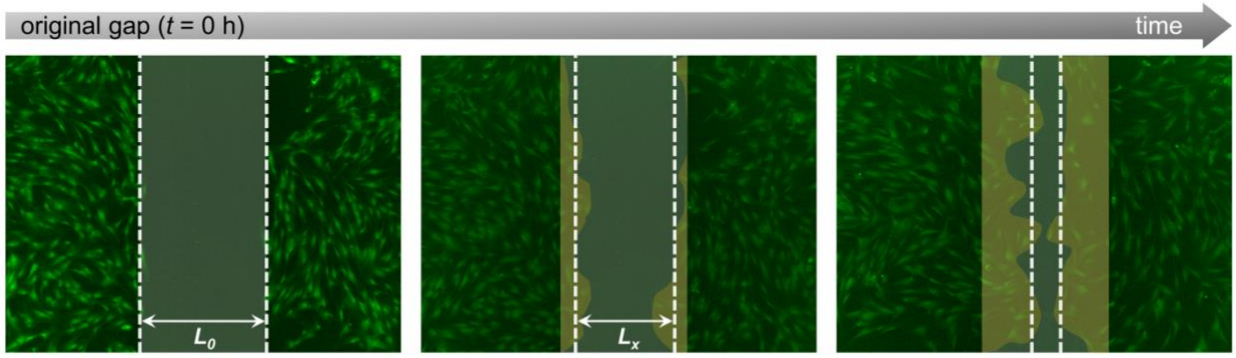

Fig. S1 Gap closure (GC) was measured as the change in gap width (L_x) in relation to the original gap width (L_0) at each time point (x) and was defined according to the following equation: $GC = \frac{L_0 - L_x}{L_0} \times 100\%$. The gap edges were defined as the recognition of the leading cells on each side of the gap with a maximum of five countable cells inside the gap that is indicated with white dotted lines in the image. Gap area filled with cells (GFC) was estimated as the fraction of the original gap area (A_0 highlighted in grey) that was evenly filled with cells at each time point (A_x highlighted in yellow) according to the following equation: $GFC = \frac{A_x}{A_0} \times 100\%$.

DNA damage

DNA damage caused by different irradiation doses presented without normalization

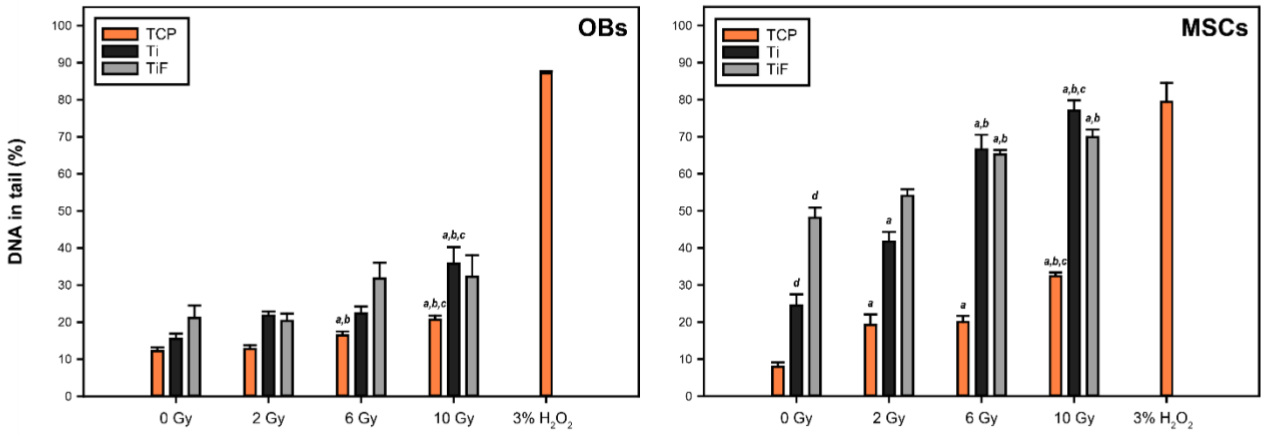

Fig. S2 Results of the comet assay presented as %DNA in comet tail (mean ± SEM, *n* = 3). For both cell types, increasing γ-radiation dose resulted in increasing DNA damage in both cell types. While similar level of DNA damage was observed for osteoblasts on the titanium surfaces (Ti and TiF) and tissue culture plastic (TCP) that were not exposed to radiation (0 Gy), the MSCs cultured on titanium showed significantly larger amount of DNA in the comet tail even without exposure to γ-radiation. The relatively high background DNA damage resulted in high %DNA in tail for the irradiated titanium samples, especially for MSCs cultured on TiF,

[a]$p \leq 0.05$ against corresponding non-irradiated negative control (0 Gy)
[b]$p \leq 0.05$ against corresponding sample irradiated with a 2 Gy dose
[c]$p \leq 0.05$ against corresponding sample irradiated with a 6 Gy dose
[d]$p \leq 0.05$ against non-irradiated negative control on TCP

DNA damage following 24 h incubation after irradiation

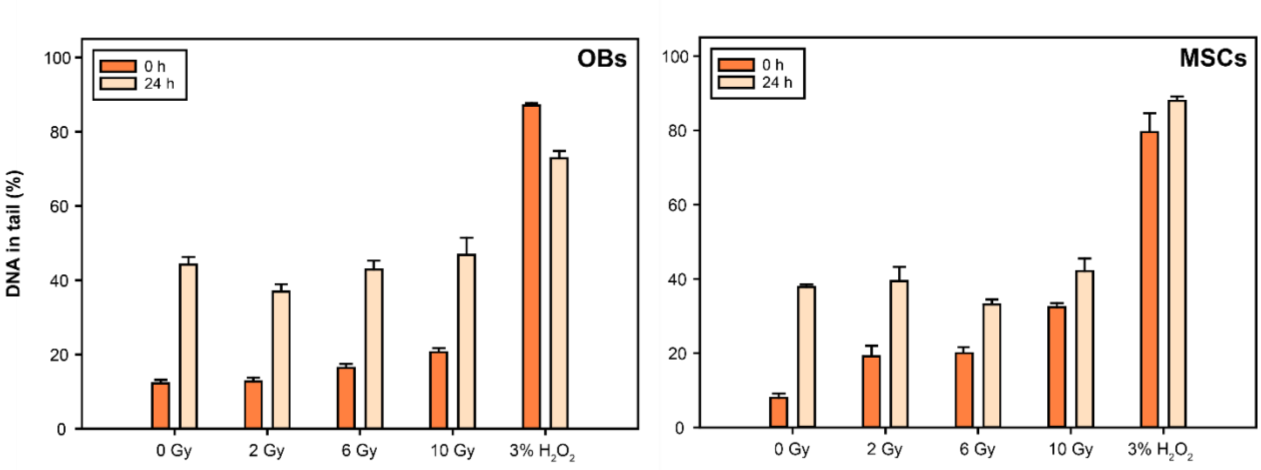

Fig. S3 DNA in comet tail observed in cells cultured on tissue culture plastic immediately after irradiation and following 24 h incubation at 37°C/5% CO_2 after irradiation (mean ± SEM, $n = 3$). As shown in Figure S2, significant dose-dependent increase in DNA damage was observed in both tested cell types immediately after exposing the cells to γ-radiation. However, no difference %DNA in tail was observed between irradiated and non-irradiated cells cultured on TCP following 24 h incubation ($p > 0.05$). While this result may indicate that the DNA damage observed immediately after irradiation is reversible, the high DNA in tail values observed for all sample groups, including the negative control group (0 Gy), following 24 h incubation renders the observation inconclusive and the absence of irreparable DNA damage in human OBs and MSCs could not be confirmed in this study. The observed high baseline DNA damage values at 24 h are not supported by the results of the migration assay or previously reported cytotoxicity results [1] and may therefore be related to the poor automatic shape detection of the used analysis software for small comets or unwanted DNA damage during sample preparation [2].

References

[1] L. Printzell, J.E. Reseland, N.F.J. Edin, J.E. Ellingsen, Effects of ionizing irradiation and interface backscatter on human mesenchymal stem cells cultured on titanium surfaces, Eur. J. Oral Sci. 127(6) (2019) 500-507.

[2] G. Speit, H. Kojima, B. Burlinson, A.R. Collins, P. Kasper, U. Plappert-Helbig, Y. Uno, M. Vasquez, C. Beevers, M. De Boeck, P.A. Escobar, S. Kitamoto, K. Pant, S. Pfuhler, J. Tanaka, D.D. Levy, Critical issues with the in vivo comet assay: a report of the comet assay working group in the 6th International Workshop on Genotoxicity Testing (IWGT), Mutat. Res. Genet. Toxicol. Environ. Mutagen. 783 (2015) 6-12.
